# Prenatal genetic diagnosis of monogenic diseases

**DOI:** 10.1515/almed-2023-0024

**Published:** 2023-03-24

**Authors:** Carmen Prior-de Castro, Clara Gómez-González, Raquel Rodríguez-López, Hada C. Macher, on behalf of the Prenatal Diagnosis Commission and the Genetics Commission of the Spanish Society of Laboratory Medicine

**Affiliations:** Servicio de Genética, Hospital Universitario La Paz, Madrid, Spain; Laboratorio de Genética, Servicio Análisis Clínicos, Consorcio Hospital General Universitario, Valencia, Spain; Departamento de Bioquímica Clínica, Hospital Universitario Virgen del Rocío de Sevilla, Sevilla, Spain; Instituto de Investigaciones Biomédicas de Sevilla, IBIS, Universidad de Sevilla, Sevilla, Spain

**Keywords:** genetic counseling, molecular techniques, monogenic diseases, prenatal genetic diagnostics, recommendations

## Abstract

Prenatal genetic diagnosis of monogenic diseases is a process involving the use of a variety of molecular techniques for the molecular characterization of a potential monogenic disease in the fetus during pregnancy. Prenatal genetic diagnosis can be performed through invasive and non-invasive methods. A distinction must be made between “NIPD” (non-invasive prenatal diagnosis), which is considered to be diagnostic, from “NIPT” (non-invasive prenatal test), which is a screening test that requires subsequent confirmation by invasive methods. The different techniques currently available aim at detecting either, previously characterized pathogenic mutations in the family, the risk haplotype associated with the familial mutation, or potential pathogenic mutation(s) in a gene associated with a diagnostic suspicion. An overview is provided of relevant aspects of prenatal genetic diagnosis of monogenic diseases. The objective of this paper is to describe the main molecular techniques currently available and used in clinical practice. A description is provided of the indications, limitations and analytical recommendations regarding these techniques, and the standards governing genetic counseling. Continuous rapid advances in the clinical applications of genomics have provided increased access to comprehensive molecular characterization. Laboratories are struggling to keep in pace with technology developments.

## Introduction

Prenatal genetic diagnosis of monogenic diseases is a process involving the use of a variety of molecular techniques for the molecular characterization of a potential monogenic disease in the fetus during pregnancy. Monogenic diseases are directly related to an alteration in a single gene. Owing to their low prevalence, these conditions are considered as rare diseases, affecting less than 5 in 10,000 inhabitants [1]. The monogenic diseases most frequently tested for prenatal diagnosis include: cystic fibrosis; Huntington’s disease; myotonic dystrophy type I; Duchene muscular dystrophy; facioscapulohumeral muscular dystrophy; Gaucher disease; Pompe disease; Friedreich’s ataxia; polycystic kidney disease; and neurofibromatosis, to name a few.

According to current laws and regulations in Spain (Order SSI/2065/2014 (BOE of November 6th) [2], prenatal diagnosis testing is indicated in the following cases:

– Upon suspicion that the fetus has a high risk of suffering from a severe genetic disease and/or his/her parents have a familial history of severe genetic disease.
– When the suspected disease is known to be caused by a specific genetic mutation and can be identified through a specific genetic test.
– Prenatal test results may lead to decisions that contribute to a tailored clinical management of pregnancy and the newborn, and guide reproductive decision-making.

Currently, prenatal genetic diagnosis can be performed either by invasive (chorionic villus sampling (CVS) or amniocentesis), or non-invasive (the starting sample is maternal peripheral blood, without it affecting fetal tissues) methods [3].

Fetal circulating DNA (cfDNA) is a small fraction of fetal DNA within the mother’s plasma cell-free DNA (3–20%). It is composed of short fragments that are less than 150 base pairs in length. cfDNA has been suggested to have an apoptotic origin and originates from fetal cells in the placenta. Fetal DNA fragments are detectable from 6 weeks of pregnancy. Its proportion increases as pregnancy progresses and are undetectable in maternal plasma at 48 h postpartum. These fragments are mostly eliminated within the first 2 h after delivery [4]. As a result, DNA from a former pregnancy cannot be detected. Fetal fraction is influenced by a range of factors, including maternal weight, toxic habits during pregnancy, maternal cancer, active maternal autoimmune disease [5] and placenta-related diseases such as preeclampsia [6], [7], [8], to name a few. Other factors affecting fetal fraction include maternal characteristics, placental-fetal characteristics, experimental factors and calculation methods [9].

Therefore, gene amplification can be performed in maternal plasma to detect fetal genetic mutations associated with monogenic diseases [10]. In the absence of these mutations in the mother, they unequivocally belong to the fetus (paternal origin or *de novo*). In this case, targeted testing is performed and considered diagnostic. These techniques are called “NIPD” (Non-Invasive Prenatal Diagnosis). Next generation sequencing (NGS) and digital PCR (dPCR) make it possible to test for maternally inherited mutations and/or X-linked recessive and dominant diseases. In this case, it is considered a screening test and is called “NIPT” (Non-Invasive Prenatal Test) and requires confirmation by invasive techniques.

Fetal DNA is detectable from the 6 weeks of pregnancy. However, the use of molecular testing in NIPD for the detection of fetal genetic mutations in maternal plasma is considered safe and diagnostic from 10 weeks of pregnancy. From this moment, the results obtained are reliable and decision-making can be performed. Nevertheless, NIPT requires confirmation by invasive techniques.

## Purpose and scope

This review provides an examination of the most relevant aspects of prenatal genetic diagnostics in monogenic diseases. A description is provided of invasive and non-invasive diagnostic molecular techniques currently used. A summary of the indications, limitations and analytical recommendations is also provided. Finally, genetic counseling guidelines are given, as it is crucial in genetic diagnosis. This review is intended to serve for consultation, help adopt an appropriate approach, and facilitate the interpretation of genetic diagnosis of monogenic diseases.

## Indications

Prenatal diagnostic testing for monogenic diseases is offered in the following cases:

– Pathogenic genetic alteration(s) have been detected in the father or mother and there is a high risk that the fetus inherits a monogenic disease:
  – The mother or the father has a genetic mutation causing a dominant autosomal monogenic disease or a dominant X-linked disease.
  – The mother is carrier of a genetic mutation causing a recessive X-linked disease. In these cases, fetal sex is determined by non-invasive techniques, whereas in males genotype is determined using invasive techniques.
  – The parents are carriers of a genetic mutation causing a recessive autosomal monogenic disease.

In some cases, gamete donation or pre-implantation genetic diagnosis (currently known as PGT: Pre-implantation genetic testing) are considered in order to conceive a healthy offspring.

– A risk haplotype associated with a monogenic disease has been identified and there is suspicion that the fetus may have inherited genetic alterations, although these alterations have not been identified.
– A brother or sister has been diagnosed with a monogenic disease caused by a *de novo* pathogenic mutation associated with a risk of germinal mosaicism.
– Ultrasound findings in the fetus suggestive of a monogenic disease.

## Molecular techniques

In prenatal diagnostic testing, a range of techniques are used as a function of the disease, type of mutation(s) under study, performance and turnaround times.

The different techniques currently available can be aimed at detecting previously characterized pathogenic genetic mutations in the family; detecting the risk haplotype associated with the familial mutation; and/or screening for potential pathogenic mutation(s) in a gene associated with a diagnostic suspicion.

Table 1 details the usefulness of the main techniques currently used in invasive and non-invasive prenatal diagnosis.

**Table 1:** Techniques used according to the type of prenatal diagnosis.

1. Invasive prenatal genetic diagnosis
Sanger sequencing
MLPA
Array-CGH/SNP array
Microsatellite testing by PCR
TP-PCR
ARMS (panels of most prevalent mutations in a specific disease)
NGS

**2. Non-invasive prenatal genetic diagnosis**

**2.1. Detection of genetic alterations not inherited from the mother (NIPD)**

Taqman real-time PCR
HRM and COLD-HRM

**2.2. Detection of genetic alterations inherited from the mother (NIPT), the father, or *de novo*** ^**a**^

Digital PCR
NGS

^a^In the case of *de novo* pathogenic variants detected in a previous son or daughter with risk of germinal mosaicism. MLPA, multiplex ligation probe amplification; TP-PCR, triplet repeat primed PCR; ARMS, amplification-refractory mutation system; NGS, next generation sequencing; HRM, high resolution melting.

### Sanger sequencing

Sanger sequencing performs the sequencing of previously amplified DNA by polymerase chain reaction (PCR) [11]. This technique is used in prenatal testing to detect point mutations (missense or nonsense variants), small deletions/duplications or small insertions. It is the technique used in invasive prenatal diagnosis to detect familial pathogenic variants in the fetus. Fetal DNA is present in maternal plasma at such low concentrations that it is below the limit of detection of the technique in non-invasive diagnosis. Therefore, this method is not used in non-invasive prenatal diagnosis.

### MLPA

MLPA (multiplex ligation probe amplification) [12] uses probes (pairs of oligonucleotides) that recognize specific regions of the target gene, generally exons. Data analysis is performed using dedicated software (Coffalyser) that greatly facilitates calculations and interpretation.

It is generally used for prenatal diagnosis of monogenic diseases caused by large deletions/duplications in a specific gene. An example is Duchenne muscular dystrophy, where 65–80% of causative mutations are deletions/duplications affecting one or more exons of the *DMD* gene [13].

As fetal DNA is found in the form of fragments in maternal plasma, the MLPA technique is not used for non-invasive diagnosis.

### Array comparative genomic hybridization (array-CGH and SNP array)

Array-CGH is based on the hybridization to equimolar amounts of sample DNA (patient) and control DNA (reference) of the same sex over DNA targets containing multiple sequences throughout the genome that are associated with a disease.

This technique enables the detection of copy number variations (CNVs) or large deletions and duplications through the genome, or regions of the genome associated with diseases. Array-CGH resolution depends on the type and design of the array. Array-CGH is very efficient in invasive prenatal diagnosis of monogenic diseases such as Duchenne muscular dystrophy [14] and cognitive impairment [15]. This technique is not used in non-invasive diagnosis, since the sample contains a mixture of fetal and maternal DNA.

CGH/SNP arrays have a high sensitivity. These arrays detect small insertions and deletions and detect heterozygosity and regions of the genome associated with imprinting.

### Microsatellite testing by PCR

Microsatellite testing is based on the determination of the number of highly polymorphic repeats of specific regions of the genome. For that purpose, polymerase chain reaction (PCR) amplification is performed using fluorescently labelled locus-specific primers. The size of amplified fragments is measured by capillary electrophoresis.

Microsatellite testing by PCR is performed when the causative mutation is unknown or testing is not possible. In these cases, indirect molecular diagnosis of the familial genetic disease is performed. The same approach is used in diseases originated by large expansions of a region of repeats (mostly trinucleotides), including myotonic dystrophy type 1 or Friedreich’s ataxia.

In these diseases, microsatellite testing by PCR is performed in conjunction with repeat primed PCR, known as triplet repeat primed PCR (TP-PCR) to test for trinucleotide repeat expansions (explained below).

As fetal DNA is found in the form of fragments in maternal plasma, this technique cannot be used in non-invasive diagnosis.

### Repeat primed PCR. Triplet repeat primed PCR (TP-PCR)

Since most repeat expansion disorders are characterized by trinucleotide expansion, focus will be placed on the TP-PCR technique.

The TP-PCR technique is a variant of PCR, with a high sensitivity and specificity for the detection of trinucleotide repeat expansions, given that one of the primers is complementary to the segment of repeating nucleotides [16]. The products obtained by TP-PCR are analyzed by capillary eletrophoresis. The presence of expansion is confirmed when a typical electrophoretic staircase or dragon tail image is obtained, with peaks progressively decreasing as the number of repeats increases.

This technique is complementary to PCR for microsatellite testing in prenatal invasive diagnosis of diseases caused by the expansion of a microsatellite, such as myotonic dystrophy type 1.

### Panels of the most prevalent pathogenic variants associated with a specific disease. Amplification-refractory mutation system (ARMS)

Considering population ancestry, the presence of a specific disease in affected individuals/families can be generally explained by the presence of pathogenic variants. There are commercially available reagents that analyze with the same assay the nucleotide positions of the most common genetic mutations in diseases such as cystic fibrosis, alpha-1-antitrypsin deficiency, or Stargardt disease. This technique is used in invasive prenatal diagnosis upon suspicion of the presence of one of these diseases in the fetus. For testing, amniocentesis or chorionic villus sampling is performed. One of the most widespread techniques used in these assays is the amplification-refractory mutation system (ARMS). This technique uses specific primers in a multiplex PCR that detects normal or wild-type alleles and mutated alleles in the gene(s) studied [17].

In the presence of a suspicious ultrasound finding (hyperechogenic bowel on fetal ultrasound/cystic fibrosis), molecular diagnostic testing is recommended [18]).

These panels do not analyze the whole gene or genes, but only the most common pathogenic variants related to the disease; therefore, they do not exclude the disease. Hence, it is important that the sensitivity of the assay for the study population and/or the residual risk of the carrier are detailed on the laboratory report, as with all assays that do not completely exclude a disease.

### Taqman real-time PCR

The Taqman probe consists of an oligonucleotide with a reporter fluorescent dye attached to the 5′ end and a quencher dye attached to the 3′ end. This probe is complementary to the target DNA. Primers are used to flank the probe-binding region. During amplification, the hybridized probe degrades and generates fluorescence as the physical distance between the fluorophore and the quencher dye increases. This is directly related to the appearance of a specific amplified product [19].

This technique has been extensively used in prenatal diagnosis and requires a specific design for each pathogenic variant and/or diagnosis. However, digital PCR (dPCR) is gaining ground over real-time PCR in many clinical scenarios.

To obtain an adequate amount of fetal DNA in non-invasive prenatal diagnosis, a larger number of amplification cycles is required for real-time PCR analysis. In this context, Taqman real-time PCR is used to detect genes that are not found in the mother, such as in fetal sex determination or when screening for the *RHD* gene in the fetus.

This technique has enough sensitivity and specificity to yield a negative result when the Y chromosome and/or the *RHD* gene is not detected in the fetus. However, when the two genes are not detected in the first sample, confirmation in a second sample is required. From 10 weeks of gestation, there is enough fetal DNA for diagnosis [20].

### Digital PCR

Digital PCR (dPCR) is based on the distribution of a target DNA across a high number of fragments resulting in individual PCR reactions [21, 22]. A total of 10,000 and 20,000 individual PCRs are performed.

This technique has a high sensitivity and detects variants at concentrations as low as 0.1% [23], which enables its use in non-invasive prenatal diagnostic testing [10] and direct quantification of the target assayed.

dPCR enables the detection of previously known causative variants inherited from the mother, the father, or *de novo* with a risk for recurrence.

The analysis of exclusively paternal sequences by dPCR requires a detection-based approach. In contrast, the study of maternal genomic regions requires calculating the relative mutation dose (RMD) in the mother´s plasma, which determines whether there is a disbalance in the fetal allelic fraction and help determine the fetal genotype [10].

This technique has been validated and demonstrated to have a high sensitivity, since it detects alleles of paternal origin with a precision of 100%. Alleles of maternal origin are detected by RMD with a precision of 96% both, in autosomal and X-linked diseases [23].

### HRM (high-resolution melting) and COLD-HRM

The HRM technique (high-resolution melting) is based on a real-time PCR in the presence of double-stranded DNA intercalating agents. These agents only emit fluorescence when they intercalate into the double strand and loss fluorescence when DNA is dissociated into single strands [24]. It differentiates DNA samples as a function of the melting curve and melting temperature (TM), thereby providing a high sensitivity and resolution to the profiles obtained by the HRM technique. Once the result of the amplified product is verified by sequencing, the HRM technique is considered to have the same sensitivity as the gold standard for the same conditions and for the specific mutation assayed.

The COLD-HRM technique is a variant of the HRM assay that uses melting temperature in a second PCR phase as the denaturalization temperature. This assay is used to detect genetic variants that are present at low concentrations in the sample. The COLD-HRM technique detects mutated DNA concentrations of 2% with respect to the wild type. Therefore, this technique is suitable for non-invasive diagnosis of fetal variants that are not inherited from the mother [25, 26].

### Massive sequencing (NGS)

NGS (next-generation sequencing) or massive sequencing is a high-throughput technology that enables the simultaneous, rapid analysis or reads of nucleotides of millions of DNA fragments. NGS detects point variants, small deletions and insertions, and detects copy number variations (CNVs) using special assays [27]. Currently, it is recommended to confirm the CNVs detected by NGS using MLPA or array.

Massive sequencing is used both, in invasive and non-invasive prenatal diagnosis but adopting completely different approaches.

#### NGS in invasive prenatal diagnosis

The diagnostic approaches used in prenatal diagnosis include:

– panel sequencing: it allows the analysis of genes associated with some diseases.
– clinical exome sequencing: sequencing of pathogenic gene-coding regions.
– whole-exome sequencing: sequencing of the regions that encode all genes of the human genome.

The use of massive sequencing in invasive prenatal diagnosis of monogenic diseases is only recommended in specific circumstances [28], [29], [30]. The American College of Medical Genetics and Genomics (ACMG) recommends that exome sequencing is only considered when diagnosis could not be confirmed by routine genetic testing (karyotype or genomic DNA array) in a fetus with multiple alterations suggestive of a genetic origin [31]. The data currently available for prenatal diagnosis by exome sequencing has been obtained in small case series. Drury et al. identified genetic alterations in 20–30% of fetuses with multiple alterations with normal results in conventional genetic studies [32].

On another note, massive sequencing has enabled the rapid sequencing of genes involved in a specific disease in fetuses where a specific phenotype has been recognized [33].

Several studies on exome sequencing demonstrate that trio sequencing (simultaneous sequencing of a sample from the fetus and the biological parents) improves diagnostic yield and expedite the interpretation of results [32, 34].

#### NGS in non-invasive prenatal diagnosis

The two main factors influencing sequence accuracy are fetal fraction, which should exceed a threshold value (4% according to guidelines), and depth of cfDNA sequencing. The reason is that a significantly greater depth is required to quantify small fractions within maternal circulating DNA, as compared to a qualitative genetic test.

##### Relative haplotype dosage analysis (RHDO)

NGS panels are used to test for maternally inherited monogenic diseases by the RHDO method [10]. Whereas relative mutation dosage (RMD) directly quantifies allelic relations for a specific variant, the RHDO technique calculates allelic gene interactions in maternal plasma for blocks of haplotypes. Hence, instead of testing for a specific variant, it detects the haplotype related to the causative variant.

In RHDO analysis, it is necessary to test for paternal and maternal haplotypes and determine the haplotype linked to the pathogenic variant (affected proband) to subsequently infer the haplotypes inherited by the fetus. Statistical power increases as the number of SNPs increases. RHDO has been reported to have a sensitivity of 100% when the percentage of fetal DNA in plasma exceeds 4% [35].

RHDO is highly effective in detecting complex genetic variants (genes with pseudogenes, large genetic deletions and pathogenic variants in repeated elements) when screening for a specific variant is not possible.

Non-invasive prenatal diagnosis by RHDO analysis is used in diseases such as Duchenne and Becher muscular dystrophy [36], cystic fibrosis or spinal muscular atrophy [37].

##### Whole-exome/genome sequencing in non-invasive prenatal diagnosis

Exome/genome sequencing in non-invasive prenatal diagnosis of monogenic diseases has not still been implanted in routine practice. However, studies are achieving to increase the efficacy of this technique [38].

**Figure 1:**
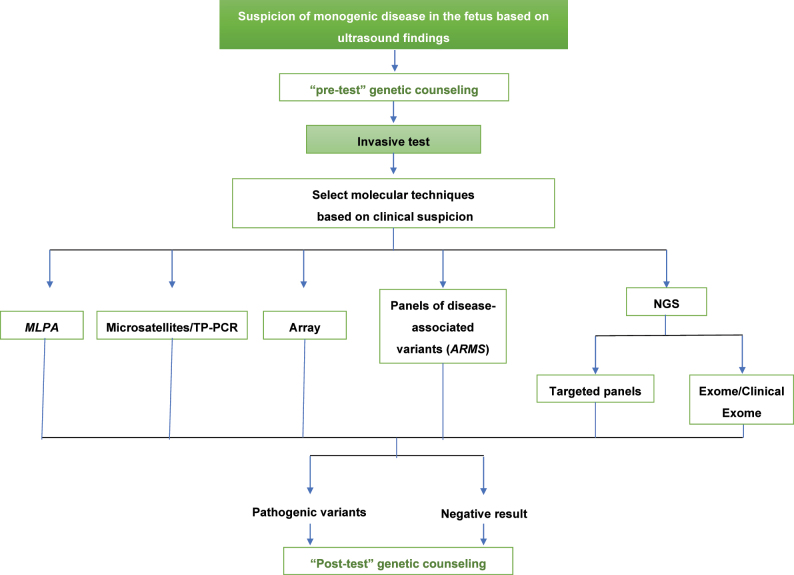
Diagnostic algorithm on suspicion of a monogenic disease in the fetus based on ultrasound findings. ARMS, amplification-refractory mutation system; MLPA, multiplex ligation probe amplification; NGS, next generation sequencing; TP-PCR, triplet repeat primed PCR*.*

**Figure 2:**
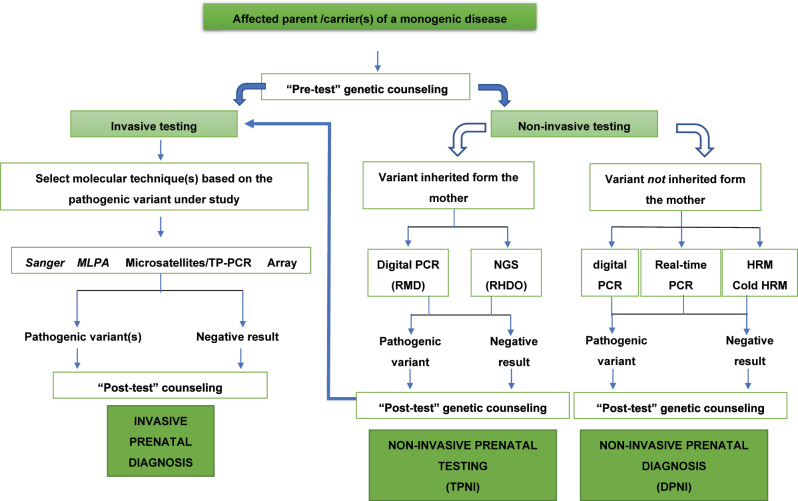
Diagnostic algorithm to exclude a monogenic disease in the fetus when the mother or the father or the two are affected by or carriers of a monogenic disease. ARMS, amplification-refractory mutation system; HRM, high resolution melting; MLPA, multiplex ligation probe amplification; NGS, next generation sequencing; NIPD, non-invasive prenatal diagnosis; NIPT, non-invasive prenatal test; RHDO, relative haplotype dosage analysis; RMD, relative mutation dosage; TP-PCR, triplet repeat primed PCR.

Figures 1 and 2 display diagnostic algorithms by genetic test indication.

## Technical limitations

### Limitations of the techniques used in invasive prenatal diagnosis

– The different techniques available will enable the detection of different types of variants (TP-PCR: repeat expansions; MLPA: large deletions and duplications, etc.). However, they cannot detect other variants, which is a limitation.
– These techniques do not detect low-level mosaicisms or contamination from maternal cells (MLPA and aCGH).
– Microsatellite testing and TP-PCR used to detect diseases caused by repeat expansions only detect the presence or absence of the pathogenic allele. However, these assays do not quantify the number of large repeat expansions [16].
– Panels of common or prevalent pathogenic variants in a disease make it possible to screen for specific variants. To expand the study and increase test sensitivity, complementary tests will be required, such as Sanger sequencing, NGS or MLPA.
– The interpretation of the variants detected is challenging. Variants of uncertain significance are frequently detected, which may cause anxiety to the family and hinder decision-making.
– Limitations of the NGS technique:
  – Poor coverage of key genes may lead to false negative results.
  – Incidental findings unrelated to the phenotype can be made [39].
  – Some pathogenic variants are not detected (large genetic rearrangements, variants in regions not included in target enrichment).

– Prenatal diagnosis requires a turnaround time of some days or weeks. As a result, the use of some molecular testing techniques is restricted to specific clinical scenarios.

### Limitations of NIPD/NIPT techniques

As these techniques use maternal plasma, the proportion of fetal DNA is the main limitation, depending on the sensitivity of the techniques used.

– For the diagnosis of a variant not inherited from the mother, the required concentration of fetal DNA in maternal plasma is 2% for real-time PCR and 0.2% for dPCR [23, 40].
– For the diagnosis of a *de novo* variant with DNA intercalating fluorophores (diagnosed based on a melting curve), conventional HRM and ColdHRM have a sensitivity of 12 and 2%, respectively [25, 26]
– When a quantitative diagnostic approach is used, the proportion of circulating fetal DNA in the mother is required to exceed 4% [5].
– However, there are factors that reduce the proportion of fetal DNA in the mother, such as maternal obesity or the presence of an inflammatory process in the mother, leading to a transient increase in maternal circulating DNA and masking fetal DNA. Other factors include the presence of a malignant neoplasm or an active autoimmune process in the mother [6], [7], [8]. Fetal fraction is also influenced by maternal characteristics, placental-fetal characteristics, experimental factors and calculation methods [9].
– The NGS technique in NIPD/NIPT:
  – It is time-consuming and expensive, since a proper read sequencing depth is necessary to be able to detect fetal DNA genotype.
  – In RHDO, a proband (affected family member) is required to establish diagnosis, but it is occasionally not available,

All limitations of the techniques used for prenatal diagnosis should be thoroughly explained during pre-test genetic counseling.

## Analytical requirements and recommendations

The laboratory performing prenatal diagnosis should be highly experienced in the diagnosis of the disease. Thus, they should use validated methods and take part in annual external quality controls.

### Guidelines for invasive prenatal diagnosis

– Diagnosis should be performed in duplicate in the fetus and, where possible, using two different methods. It is recommended that, on receipt in the laboratory, the amniotic fluid or chorionic villus sample is divided into two aliquots for subsequent separate processing.
– It should be verified that the fetal sample is free of maternal contamination.
– Processing of the samples of the index case and the parents should be performed in parallel to the processing of the fetal sample, whenever possible.
– For most tests, results should be reported within a week from receipt of the sample in the laboratory.
– The report should be clear and concise, and should detail [41]:
  – that the presence of maternal contamination has been ruled out.
  – the clinical significance of the molecular result.
  – Whenever indirect diagnosis is performed, it is important to report the risk on the laboratory report.

### Guidelines for non-invasive prenatal diagnosis

Determination of genetic alterations in circulating DNA should be performed in very strict pre-analytical conditions.

– When massive sequencing of circulating DNA in blood is being performed, it is recommended to use plasma (shipped in an EDTA tube). Plasma (shipped in an EDTA tube) and serum can be used for the diagnosis of genes not found in maternal DNA, as in the case of RHD, and for sex determination. Serum is used for dPCR and HRM, although plasma can also be used [42].
– It is important that the tube is received in the laboratory within 4–6 hours from collection, unless a special tube is used and cell integrity is protected [42].
– Plasma and extracted circulating DNA should be stored at −80 °C and −20 °C, respectively, to avoid freezing and thawing cycles [42].
– It is recommended that 100 to 400 pb-length fragments are obtained, as they preferentially select fetal DNA (there are specific kits available for the extraction of circulating DNA) [42].
– The fraction of fetal DNA should exceed 4% [5] when massive sequencing is being performed for quantification and invasive prenatal diagnostic testing is recommended if the fraction of fetal DNA does not exceed 4%. When real-time PCR techniques are used, a lower fetal fraction can be used, as it is a qualitative diagnosis, with 2% being accepted for ColdHRM [25, 26] and 0.2% for dPCR [23].
– In NGS studies, to prevent false negative results due to very low concentrations of circulating fetal DNA, it is recommended to identify paternally inherited alleles or demonstrate the presence of fetal DNA and perform quantification.
– If the placenta causes preeclampsia, an invasive fetal diagnostic technique should be used.
– The presence of mosaicisms confined to the placenta should be considered, since the fetal component originates from placental cells and may yield false positive or inconclusive results.
– To prevent false positive results, the presence of an empty gestational sac from a disappeared affected embryo (undetected), known as “vanishing or disappearing twin” should be excluded by ultrasound examination.
– Potential sources of foreign DNA in the mother, as in organ transplant recipients, should be excluded.
– NIPD is not recommended for multiple pregnancy, except in case of inconsistent ultrasound findings [5].

The prenatal diagnostic techniques currently available are varied and increasingly complex, which poses new analytical, legal and ethical challenges.

## Genetic counseling

Definitions and details about prenatal genetic counseling are provided in the Spanish Law on Biomedical Research [43]; *American Society of Human Genetics* guidelines (https://www.acmg.net/ACMG/Medical-Genetics-Practice-Resources/Practice-Guidelines.aspx); and the Council of Europe Convention on Human Rights and Biomedicine [44], to name a few (https://www.nsgc.org/page/specialty-areas).

During prenatal genetic counseling, families are provided with information on potential congenital defects in their future children, i.e. anatomical, structural, functional or molecular alterations at birth (albeit they may manifest later in life), which may be visible and/or present in the internal organs of the newborn. Families are also explained that congenital defects may be familial or occur sporadically, may be inherited or not, and they may present as a sole developmental defect or as part of a range of defects. Future parents are referred to prenatal genetic counseling upon suspicion that the fetus may suffer from a genetic disease or that the parents may pass a genetic disease on to their offspring. Referral to a prenatal genetic counselor may be requested at preconception, preimplantation and/or during pregnancy. The purpose of genetic counseling is to diagnose or predict the presence of a genetic disease in the fetus, which is frequently associated with severe disabilities. There must be robust evidence available demonstrating that the genetic alteration is pathogenic and causative of disease.

The genetic counselor must ensure that the couple understands the information provided, so that they can freely make an informed decision. Their role is to provide guidance rather than direction. Genetic counseling requires compliance with all applicable laws and regulations [2, 43], [44], [45].

Genetic test results must be provided in a rapid, clear way and interpreted carefully on the basis of the latest scientific evidence available on the date of consultation. In the prenatal period, fetal phenotypical data can only be obtained by ultrasound. Results are inconclusive but help interpret and address the results of diagnostic genetic testing.

Prior to genetic testing, at least a genetic counseling session is required (“pre-test” counseling). After testing, another session is necessary to provide and explain the results obtained (“post-test” counseling).

Genetic testing laboratories are recommended to comply with and hold ISO 17025, ISO 15189 and ISO 9001 certification. Further guidelines and recommendations are provided by the Asociación Española de Genética Humana or the OECD [46], [47], [48], [49].

### Pre-test genetic counseling

During the pre-test genetic counseling visit, the counselor:

1. Explains the reason why the family has been referred to genetic counseling
2. Collects personal data, along with information about familial medical history, and clinical, analytical and genetic reports provided by the requesting person/family in relation to the reason for referral. Constructing a genealogical tree as long as it is possible is essential
3. Provides information on the diagnostic strategy and explains the purpose of genetic testing, its diagnostic value and the type of sample to be obtained. The counselor also explains the advantages of genetic testing and the possibility that it confirms diagnosis, prognosis and treatment. It is essential to inform on the precision and limitations of the indicated test and describe other alternative tests available. The patient must be aware of the potential results that may be obtained. They must also know whether the sole causative agent of the disease can be identified or not. They must be made aware that results of uncertain significance may be obtained, which implications are unknown [50]. Information must be provided that an incidental finding of other diseases unrelated to the diagnostic suspicion may be made. The counselor also explains the potential implications that the results of genetic testing may have for other family members.
4. Informed consent. The pregnant woman declares that she understood the information received and that participation is voluntary, and agrees to undergoing the indicated genetic test. She also states her decision about sample storage and on whether she wants to be informed or not on any incidental findings and/or results of uncertain significance that may be obtained

It has been agreed that consent must be provided in written and recorded on the medical record of the patient. The counselor must be aware that it is a legal and ethical obligation towards patients in genetic counseling visits [51].

### Post-test genetic counseling

During the post-test genetic counseling session, the pregnant woman and/or her partner are informed on the results of the genetic tests performed. Results must be reported in clear and simple terms. The counselor explains their implications for the newborn and their family, the risk of recurrence in the offspring, and the primary or secondary prenatal or postnatal prevention measures available.

Apart from the explanation of the genetic results obtained, fetal analytical and clinical data in relation to the genetic test should also be recorded. Segregation of genetic variants in affected and unaffected family members may be required or useful.

It is essential that genetic counselors coordinate clinical care or surveillance by as many specialists as it is necessary for the fetus, the future offspring, progenitors and other family members, to ensure that each individual situation is managed appropriately.

There is increasing evidence of the important role that early genetic diagnosis plays. With the advent of new therapeutic practices, genetic variants can be totally corrected, or at least their manifestations can be moderated.

## Conclusions

Prenatal genetic diagnosis of monogenic diseases has traditionally been performed by invasive methods. However, it can be carried out using a sample of maternal peripheral blood, thereby preventing any damage to fetal tissues. Non-invasive techniques prevent maternal stress and the associated risk of miscarriage, which is accepted to range from 0.11% post-amniocentesis and 0.22% post-chorionic villus sampling (CVS) [52] to 0.35% post-amniocentesis and post CVS [53]. In prenatal diagnosis, the molecular approach used should be tailored to the particular situation. Therefore, it is important that specialists are aware of the techniques available and their limitations to ensure that an appropriate approach is adopted for prenatal diagnosis. Progress in massive sequencing techniques and dPCR facilitates the diagnosis of monogenic diseases. Laboratories must ensure that they keep pace with continuous advances in genetic testing.

Genetic counseling by a trained professional should always accompany prenatal genetic diagnosis. This practice enables informed decision-making and help individuals being tested understand the implications of the genetic results obtained.
